# Transmembrane protein 170B is a novel breast tumorigenesis suppressor gene that inhibits the Wnt/β-catenin pathway

**DOI:** 10.1038/s41419-017-0128-y

**Published:** 2018-01-24

**Authors:** Mengwei Li, Yanzhen Han, Haoze Zhou, Xin Li, Chenyu Lin, Erhao Zhang, Xiaowei Chi, Jialiang Hu, Hanmei Xu

**Affiliations:** 1 The Engineering Research Center of Peptide Drug Discovery and Development, China Pharmaceutical University, Nanjing, 210009 China; 2 State Key Laboratory of Natural Medicines, Ministry of Education, China Pharmaceutical University, Nanjing, 210009 China

## Abstract

The identification of specific drug targets guides the development of precise cancer treatments. Compared with oncogenes, tumor suppressor genes have been poorly studied in the treatment of breast cancer. We integrate the microRNA expression array from GEO (Gene Expression Omnibus) and TCGA (The Cancer Genome Atlas) databases in clinical breast cancer tissues, and find that miR-27a is significantly upregulated and correlated with poor survival outcome and tumor progression. Transmembrane protein 170B (TMEM170B), a new functional target of miR-27a, is identified via target prediction and experimental validation, suppressing breast cancer proliferation, metastasis, and tumorigenesis. Furthermore, TMEM170B overexpression promotes cytoplasmic β-catenin phosphorylation, resulting in the inhibition of β-catenin stabilization, reduction of nuclear β-catenin levels and downstream targets expression. Clinically, TMEM170B or β-catenin expression is significantly correlated with overall survival ratio in breast cancer patients. Thus, these results highlight TMEM170B as a novel tumor suppressor target in association with the β-catenin pathway, which may provide a new therapeutic approach for human breast cancer therapy.

## Introduction

Breast carcinoma (BRCA) remains one of the top three most frequently diagnosed forms of carcinomas and the most aggressive type of tumors in women. It has been estimated that BRCA accounted for 29% of all newly diagnosed cancer cases in 2016^1,2^. Despite the potential of clinical combination therapy, we must acknowledge that poor prognosis and high recurrence rates seriously restrict clinical treatments. Therefore, there is an urgent need to discover novel therapeutic targets and prognostic markers for treatment of BRCA^3,4^. Carcinogenesis is a complex process that involves oncogenes and suppressor genes^5,6^. Currently, numerous breast cancer oncogenes have been reported and extensively characterized^7,8^, whereas relatively few studies have focused on tumor suppressor genes. Thus, novel suppressor genes and the molecular pathways underlying BRCA progression and metastasis need to be identified and validated.

MicroRNAs (miRNAs) are a class of small noncoding RNAs that can bind to the 3′-untranslated regions (3′-UTRs) of target messenger RNAs (mRNAs), leading to mRNA degradation and translational suppression^9^. Emerging evidence has indicated that some miRNAs are abnormally expressed, and may function as onco-miRNAs or suppressor miRNAs in human cancers^10,11^. miRNA-27a belongs to the microRNA-27 family, and it is located at chromosome 19^12^. New evidence indicates that miRNA-27a is abnormally expressed and regulates epithelial–mesenchymal transition and metastasis in various cancers^13–15^. In addition, previous studies have shown that miR-27a is an activator of the Wnt signaling pathway^16^. The important role of Wnt/β-catenin signaling in breast cancer metastasis and the stem cell process has been confirmed^17^. Thus, these results have prompted the search for the direct targets of miR-27a and an understanding of how the molecular regulation of the Wnt/β-catenin pathway occurs.

In this study, using bioinformatics analysis of different public databases (including mRNA and miRNA sequencing data) combined with experimental assays, we confirmed that the transmembrane protein TMEM170B, is a novel direct target of miR-27a, and that it is significantly downregulated in breast cancer. TMEM170B, a member of TMEM170 family is comprised of 132 amino acids, and the sequences are highly conserved from invertebrates to mammals. However, its function in biological processes, especially in cancer progression, is unknown. Thus, understanding the function of TMEM170B and the signaling pathway connected with the miR-27a/TMEM170B axis are essential for the development of more effective strategies for treating breast cancer malignancy. We have also analyzed the association of TMEM170B expression with the clinical overall survival (OS) ratio and associated pathological features to explore its prognostic and therapeutic value in preventing breast cancer progression.

## Results

### TMEM170B, a direct target of miR-27a, is downregulated in breast cancer

**Fig. 1:**
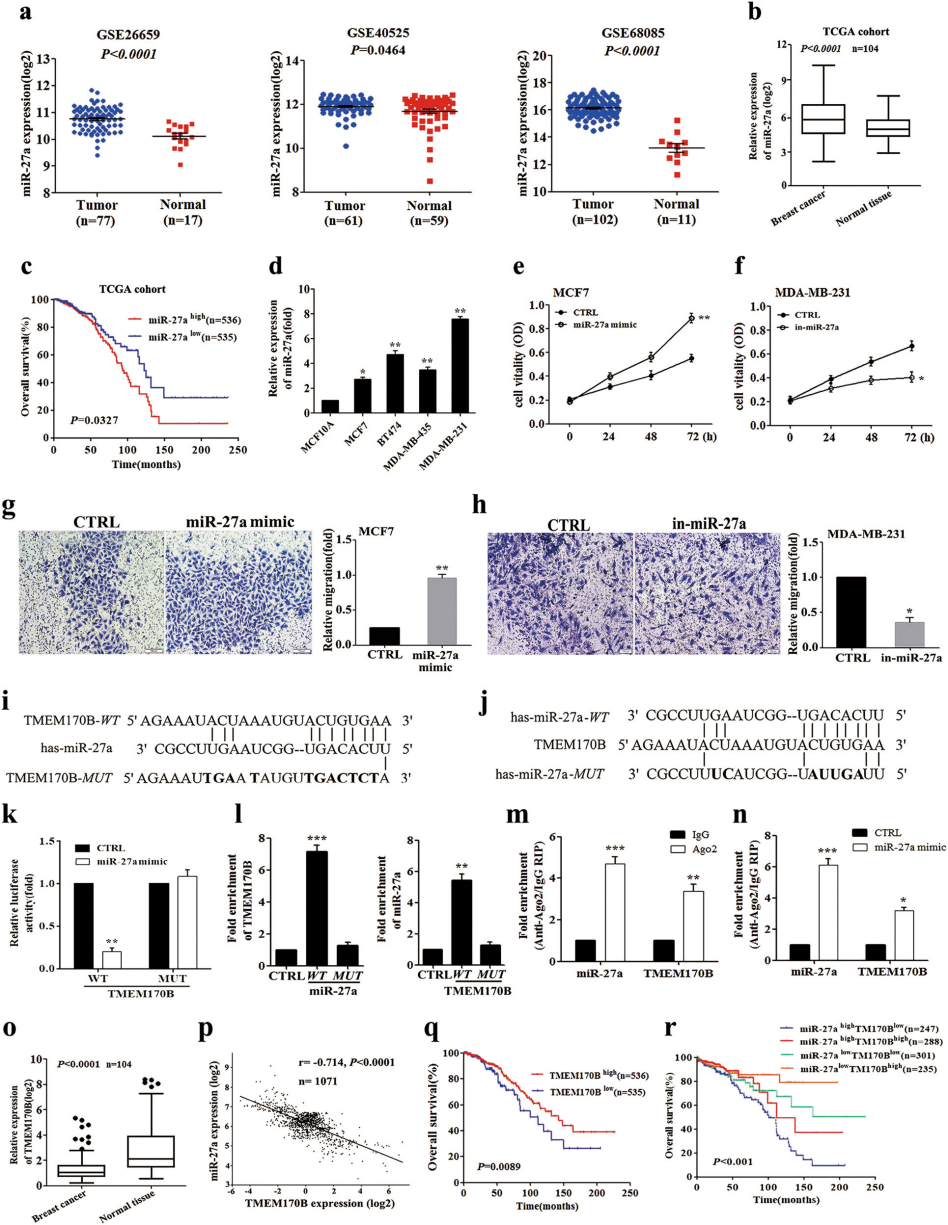
TMEM170B as a direct target of miR-27a, is downregulated in breast cancer **a** Expression profiles of miRNA-27a in primary breast cancer and normal breast tissues of published GEO profiles (NCBI/GEO/GSE 26659/40525/68085). **b** Differential expression of miR-27a in breast cancer and paired adjacent noncancerous tissues from the TCGA database. **c** Kaplan–Meier survival analysis of the overall survival in 1071 BRCA patients with different miR-27a expression. **d** The miR-27a expression of the breast cancer cells were detected by real-time PCR. **e**, **f** Cell proliferation of MCF7 cells transfected with miR-27a mimic (**e**) or MDA-MB-231 cells with miR-27a inhibitor (**f**). **g**, **h** Cell migration assays of MCF7 cells transfected with miR-27a mimic (**g**) or MDA-MB-231 cells with miR-27a inhibitor (**h**). **i**, **j** The schematic sequence of constructs used in the dual-luciferase reporter assay and the potential miR-27a binding site with TMEM170B. **k** The luciferase reporter activity in 293T cells transfected with miR-27a mimic or control and luciferase reporters containing TMEM170B wild type or mutant. **l** RNA pull-down analysis of relative fold enrichment of TMEM170B using biotin-labeled miR-27a or fold enrichment of miR-27a using biotin-labeled TMEM170B. **m**, **n** Relative enrichment of miR-27a and TMEM170B in the RIP assay using anti-Ago2 antibody. **o** Differential expression of TMEM170B between breast cancer and paired adjacent noncancerous tissues in the TCGA database. **p** The correlation of TMEM170B and miR-27a expression in TCGA cohort. **q**, **r** Kaplan–Meier survival analysis of the overall survival in 1071 BRCA patients with different TMEM170B expression (**q**) or miR-27a/TMEM170B expression patterns (**r**). All of the data show mean ± S.E.M. *P < 0.05 vs. CTRL, **P < 0.01 vs. CTRL, ***P < 0.001 vs. CTRL

We first analyzed multiple miRNA expression profiling data sets of breast tumor samples (NCBI/GEO/GSE 26659/40525/68085), and we found that miR-27a was significantly overexpressed in breast cancer compared with normal breast tissues (Fig. 1a). In the TCGA cohort, miR-27a expression was also upregulated in the BRCA tissues compared to paired normal breast tissues (*n* = 104, Fig. 1b). Moreover, Kaplan–Meier analysis revealed that the BRCA patients with high miR-27a levels had worse OS time (15% vs. 37%, *P* < 0.05, Fig. 1c). Real-time PCR analysis showed that miR-27a expression was markedly increased in the breast cancer cells compared with the normal mammary cells MCF10A (Fig. 1d and Supplementary Fig. 1a). To further examine the role of miR-27a in breast cancer, we performed overexpression and knockdown functional assays in breast cancer cells using miR-27a mimic and inhibitor (Supplementary Fig. 1b and c). The overexpression of miR-27a significantly promoted the proliferation, migration, and invasion in MCF7 cells (low metastatic cells, Fig. 1e, g and Supplementary Fig. 1d), whereas the knockdown of miR-27a significantly attenuated the cell proliferation, migration, and invasion in MDA-MB-231 cells (high metastatic cells, Fig. 1f, h and Supplementary Fig. 1e). All of these results showed that the miR-27a levels were positively associated with breast cancer cell proliferation, migration, and invasion.

Over the past decade, computational target prediction programs including MiRanda (http://www.microrna.org/), TargetScan (http://www.targetscan.org/archives.html), PicTar (http://pictar.bio.nyu.edu), RNAhybrid (http://bibiserv.techfak.uni-bielefeld.de/), and StarBase v2.0 (http://starbase.sysu.edu.cn/) have provided putative binding sites for miRNAs^18–20^. However, target prediction still remains a challenge due to the unavailability of analytical programs. Full combination with experimental validation is important to increase the prediction accuracy. We performed bioinformatics analysis of the data from the miRDB, Targetscan, Pictar, and miRanda websets to explore the molecular mechanism of miR-27a as an oncogenic molecule in breast cancer, and found that six genes are overlapped, namely *TMEM170B*, *GPAM*, *PPAP2B*, *ST6GALNAC3*, *EYA1*, and *PPARG* (Supplementary Fig. 1f). To determine whether miR-27a targets these genes, we generated luciferase reporter plasmids that harbor miR-27a target sequences in the 3′-UTR of these genes. By using these luciferase reporter plasmids, we found that miR-27a mimic did not significantly inhibit the activities of 3′-UTR luciferase reporters of *GPAM*, *PPAP2B*, *ST6GALNAC3*, *EYA1*, or *PPARG* gene (Supplementary Fig. 1g–k). However, expression of miR-27a mimic caused significantly strong inhibition of the activities of 3′-UTR luciferase reporter of *TMEM170B* gene, which did not occur with the mutant (*MUT*) reporter vector (Fig. 1i, k). The sequence of the miR-27a-binding site within *TMEM170B* 3′-UTR was depicted by the Targetscan webset (Supplementary Fig. 1l). The same seed sequence is present in the highly conserved TMEM170B protein sequence of eukaryotic phyla (Supplementary Fig. 1m), which suggests the importance of TMEM170B in different species.

In addition, the specific interaction between miR-27a and TMEM170B was further verified using the biotin-avidin pull-down system (Fig. 1j). TMEM170B was pulled down by the biotin-labeled miR-27a, but not the miR-27a mutant. In a reciprocal manner, miR-27a was also precipitated by wild-type TMEM170B but not by the TMEM170B mutant (Fig. 1l). Moreover, RNA immunoprecipitation (RIP) assays showed that miR-27a directly bound to TMEM170B mRNA (Fig. 1m, n) in the RNA-induced silencing complex (RISC). Together, these results suggest that miR-27a downregulates TMEM170B expression by directly targeting its 3′-UTR.

The TMEM170B expression in the TCGA cohort (*n* = 104) was also downregulated in the BRCA tissues (Fig. 1o). Consistently, a significantly negative correlation was observed between TMEM170B and miR-27a in the BRCA tumors (*r* = −0.714, *P* < 0.0001, Fig. 1p). In addition, using StarBase 2.0, we found that TMEM170B expression in the BRCA samples with low miR-27a levels was significantly increased compared with those with high miR-27a levels (Supplementary Fig. 1n). Kaplan–Meier analysis revealed that the BRCA patients with low TMEM70B levels had a lower OS ratio (23% vs. 40%, *P* < 0.01, Fig. 1q). Moreover, the BRCA patients with the low TMEM70B and high miR-27a levels had the worst OS ratio (10%, *P* < 0.001, Fig. 1r). Taken together, these results suggest that downregulation of TMEM70B may contribute to the malignant progression of breast cancer.

Intriguingly, TMEM170B levels were downregulated by miR-27a mimic, as well as upregulated by miR-27a inhibitor (Supplementary Fig. 1o and p). However, no significant difference in miR-27a expression was detected after ectopic expression or deficiency of TMEM170B (Supplementary Fig. 1q and r). Collectively, these results revealed that TMEM170B was a direct target of miR-27a, but there was no feedback regulation between TMEM170B and miR-27a.

### TMEM170B exerts an inhibitory effect on breast cancer growth

**Fig. 2:**
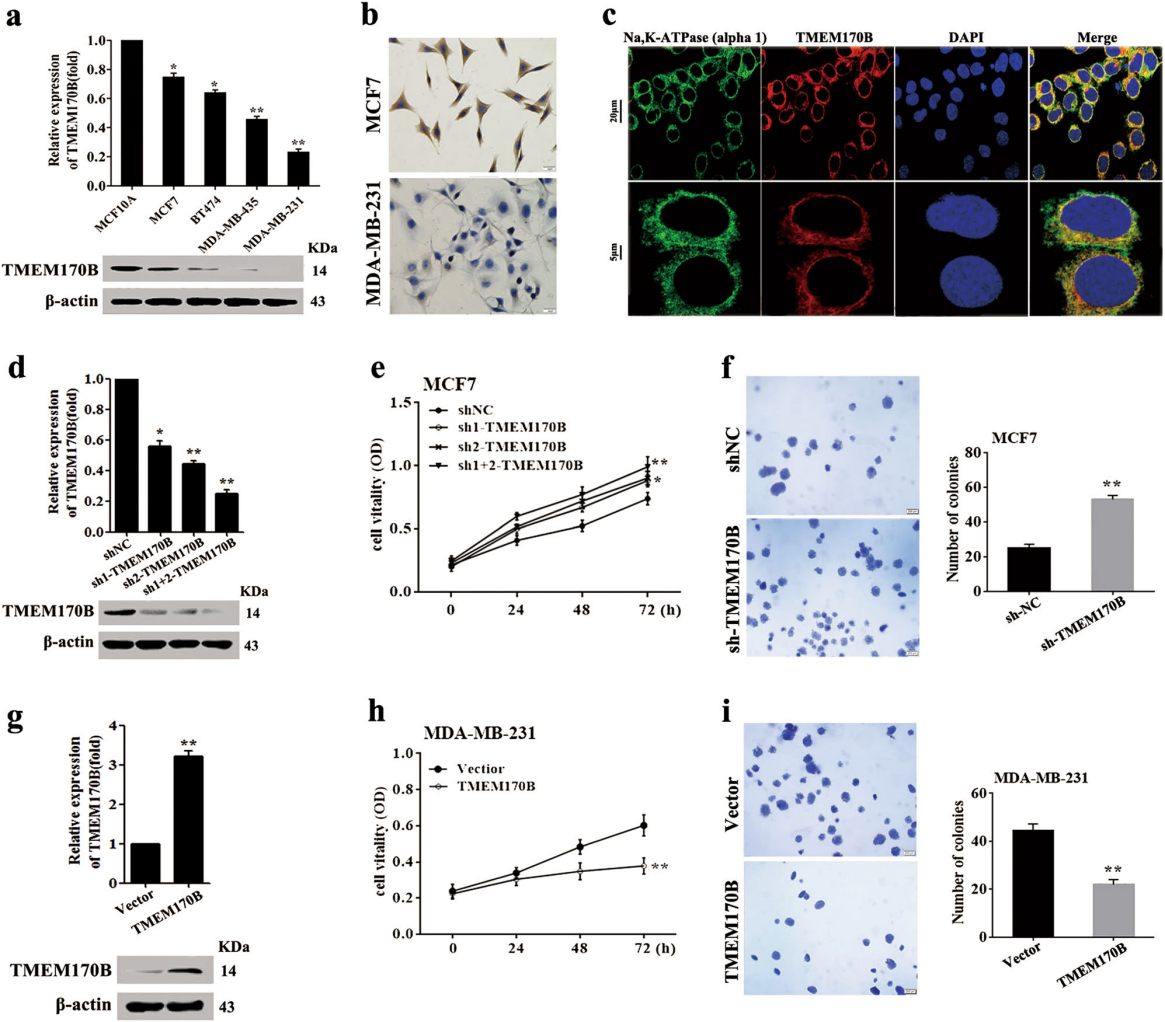
TMEM170B suppressed breast cancer cell proliferation **a** The TMEM170B expression in breast cancer cell lines was detected by real-time PCR and immunoblotting. **b** Immunocytochemical staining analysis of the cell membrane localization of TMEM170B in MCF7 and MDA-MB-231 cells. **c** The subcellular localization of TMEM170B with Na,K-ATPase (alpha 1), a membrane marker of plasma membrane, was analyzed by immunostaining (upper: scale bar, 20 μm; lower: scale bar, 5 μm). **d**, **e** Relative expression of miR-27a in the indicated MCF7 cell clones (**d**) and MDA-MB-231 cell clones (**e**). Cell proliferation (**f**) and soft agar assays (**g**) were performed on MCF7 cells with TMEM170B knockdown. Cell proliferation (**h**) and soft agar assays (**i**) were performed on MDA-MB-231 cells with overexpressing TMEM170B. Values are shown as mean ± S.E.M. from three independent experiments repeated in triplicates. *P < 0.05 vs. CTRL, **P < 0.01 vs. CTRL

We first examined the TMEM170B levels in different breast cancer cells by real-time PCR (RT-PCR) and immunoblotting to investigate the functional roles of TMEM170B in breast cancer progression (Fig. 2a). To determine the specificity of the TMEM170B antibody, we conducted immunoblotting (Supplementary Fig. 2a and b) and IHC (immunohistochemistry) assays (Supplementary Fig. 2c) with different concentrations of TMEM170B antibody, and using human serum albumin as negative control, the results showed that the antibody had highly specificity at the appropriate concentration. Consistent with the RT-PCR results, IHC analysis (Fig. 2b) revealed that the TMEM170B expression levels in MCF7 cells (low metastatic cells) were dramatically higher than that in MDA-MB-231 cells (highly metastatic cells). In addition, we analyzed the subcellular localization of TMEM170B with immunostaining and subcellular protein fractionation as described in the previous *MUC1* report^21^. We found that TMEM170B mainly colocalized on the cell membrane (Fig. 2c), which was labeled by the Na,K-ATPase (alpha 1) as shown previously^22^. A similar observation (Supplementary Fig. 2d) was also made by immunoblotting of nuclear, cytoplasmic, and membrane fractions with internal controls. Subsequently, we established stable models of TMEM170B knockdown in MCF7 cells (Fig. 2d), and stable models of TMEM170B overexpression in MDA-MB-231 cells (Fig. 2g). Knockdown of TMEM170B significantly induced the cell proliferation and colony-formation ability of breast cancer (Fig. 2e, f), whereas the overexpression of TMEM170B significantly impeded the breast cancer cells proliferation and colony-formation ability in vitro (Fig. 2h, i).

**Fig. 3:**
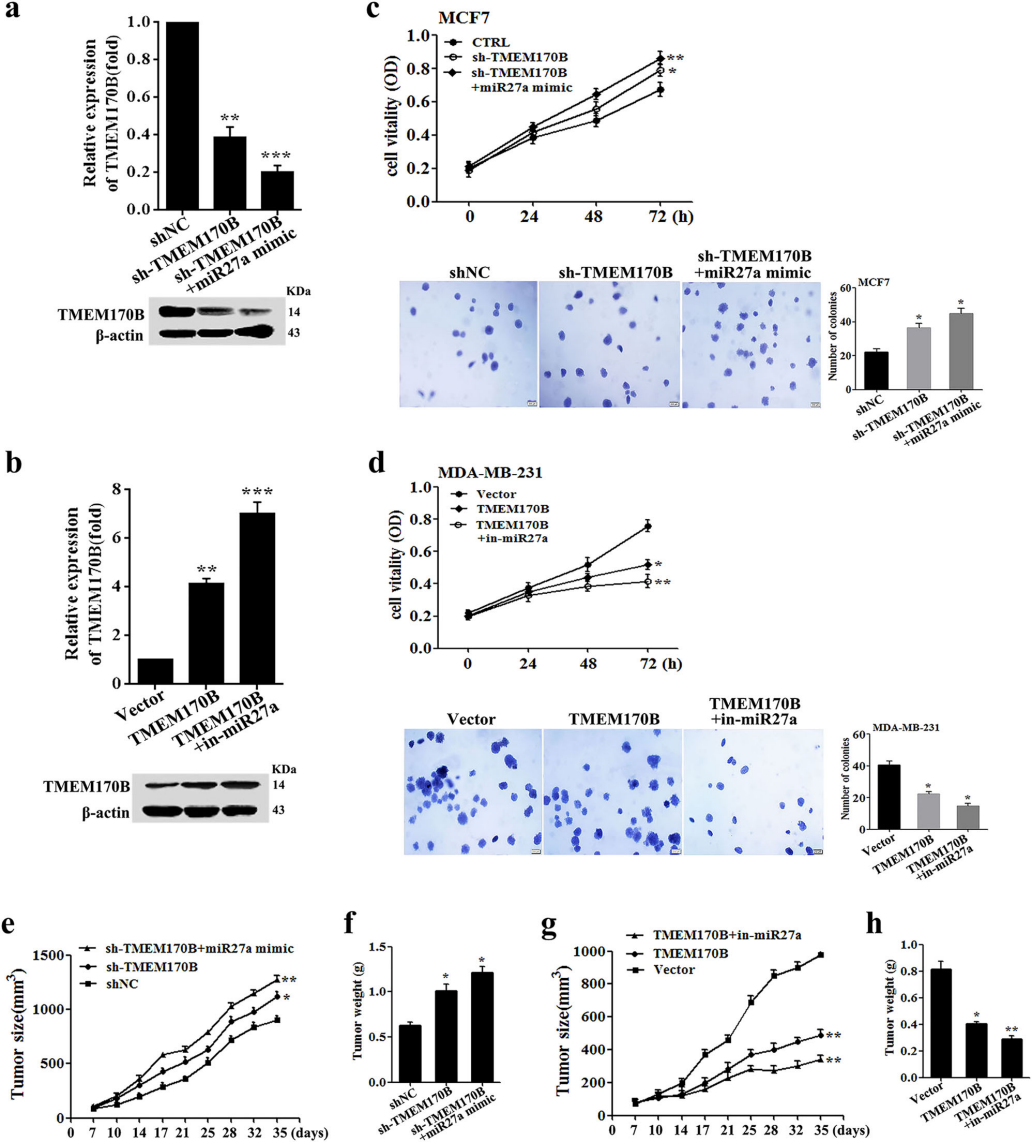
miR-27a as a negative regulator of TMEM170B in breast cancer growth **a**, **b** The TMEM170B expression in the indicated MCF7 cell clones (**a**, *n*** = **4) and MDA-MB-231 cell clones (**b**, *n*** = **4). Cell proliferation and soft agar assays (**c**) were performed on the indicated MCF7 cell clones (*n*** = **4). Cell proliferation and soft agar assays (**d**) were performed on the indicated MDA-MB-231 cell clones (*n*** = **4). Tumor volume (**e**) and tumor weight (**f**) of the mice in each group (*n* = 5) following orthotopic implantation with the indicated MCF7 cells. Tumor volume (**g**) and tumor weight (**h**) of the mice in each group (*n*=5) following orthotopic implantation with the indicated MDA-MB-231 cells. Values are shown as mean ± S.E.M. *P < 0.05 vs. CTRL, **P < 0.01 vs. CTRL

We then performed rescue experiments to determine whether miR-27a can functionally target TMEM170B, and to further demonstrate the importance of the miR-27a/TMEM170B axis in the progression of breast cancer. The co-transfected efficiency was evaluated by RT-PCR and immunoblotting (Fig. 3a, b). The functional assays showed that miR-27a mimic specifically aggravated the cell proliferation and colony-formation ability, which were promoted by TMEM170B knockdown (Fig. 3c). In contrast, miR-27a inhibitor further attenuated the cell proliferation and colony-formation ability, which was decreased by TMEM170B overexpression (Fig. 3d). Moreover, TMEM170B knockdown significantly promoted tumorigenicity in vivo (Fig. 3e, f), whereas TMEM170B overexpression notably inhibited tumorigenicity in vivo (Fig. 3g, h). Consistent with the in vitro results, miR-27a negatively regulated the suppressive function of TMEM170B (62% vs. 45% for MCF7 xenografts, *P* < 0.01; 62% vs. 41% for MDA-MB-231 xenografts, *P* < 0.01, Fig. 3e–h), indicating that the miR-27a/TMEM170B axis may be a critical factor that drives tumorigenesis in breast cancer.

### TMEM170B suppresses the migration and invasion ability in breast cancer

**Fig. 4:**
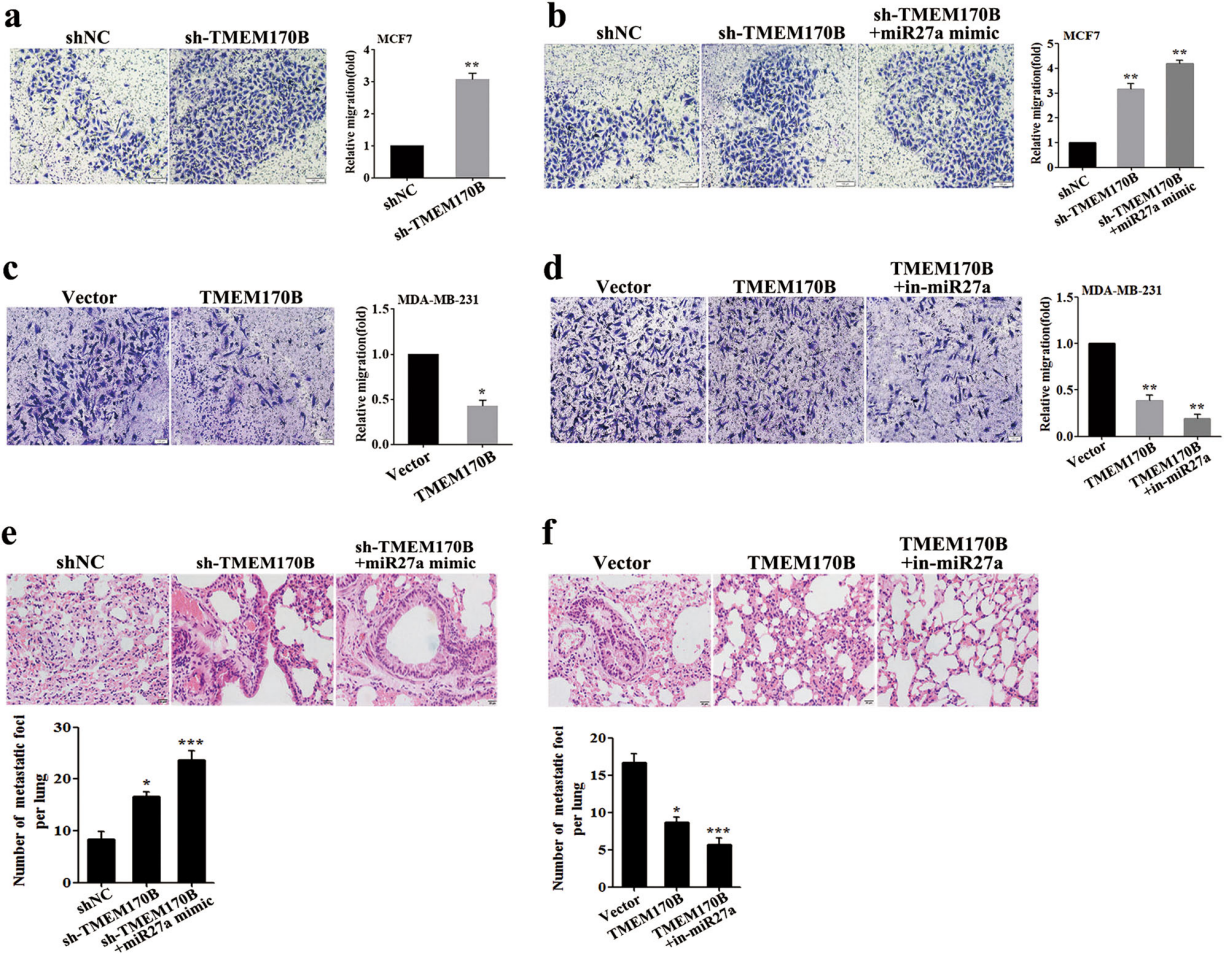
TMEM170B exerted an inhibitory effect on breast cancer cell migration and invasion **a**, **b** Cell migration assay of MCF7 cells with TMEM170B knockdown without (**a**) or with (**b**) miR-27a mimic. **c**, **d** Migration assays of MDA-MB-231 cells with overexpressing TMEM170B without (**c**) or with (**d**) the miR-27a inhibitor. Lung metastasis of the mice in each group (*n* = 5) following orthotopic implantation with the indicated MCF7 (**e**) or MDA-MB-231 cells (**f**). Data are presented as mean ± S.E.M. from three independent experiments repeated in triplicates. *P < 0.05 vs. CTRL, **P < 0.01 vs. CTRL, ***P < 0.001 vs. CTRL

We next investigate the effect of TMEM170B on the cell migration and invasion ability to further understand the tumor-suppressive role of TMEM170B. Knockdown of TMEM170B significantly promoted the cell migration and invasion ability (Fig. 4a and Supplementary Fig. 3a), whereas miR-27a mimic further augmented the cell migration, invasion, and wound-healing mediated by TMEM170B knockdown in MCF7 cells (Fig. 4b and Supplementary Fig. 3b and e). In addition, overexpression of TMEM170B significantly slowed the cell migration and invasion of breast cancer cells in vitro (Fig. 4c and Supplementary Fig. 3c), while miR-27a inhibitor prevented the cell migration, invasion, and wound-healing mediated by TMEM170B overexpression in MDA-MB-231 cells (Fig. 4d and Supplementary Fig. 3d and f). Furthermore, miR-27a mimic and significantly increased the number of metastatic lung nodules in the TMEM170B knockdown groups (Fig. 4e), and miR-27a inhibitor significantly decreased the number of metastatic lung nodules in the TMEM170B overexpression groups in vivo (Fig. 4f). Gain and loss function of TMEM170B had no marked impact on other tissues (Supplementary Fig. 4a and b). These findings suggested that TMEM170B has an inhibitory effect on breast cancer cell migration and invasion in vitro and colony metastasis in vivo.

### TMEM170B performs as an endogenous inhibitor of the Wnt/β-catenin pathway in breast cancer

**Fig. 5:**
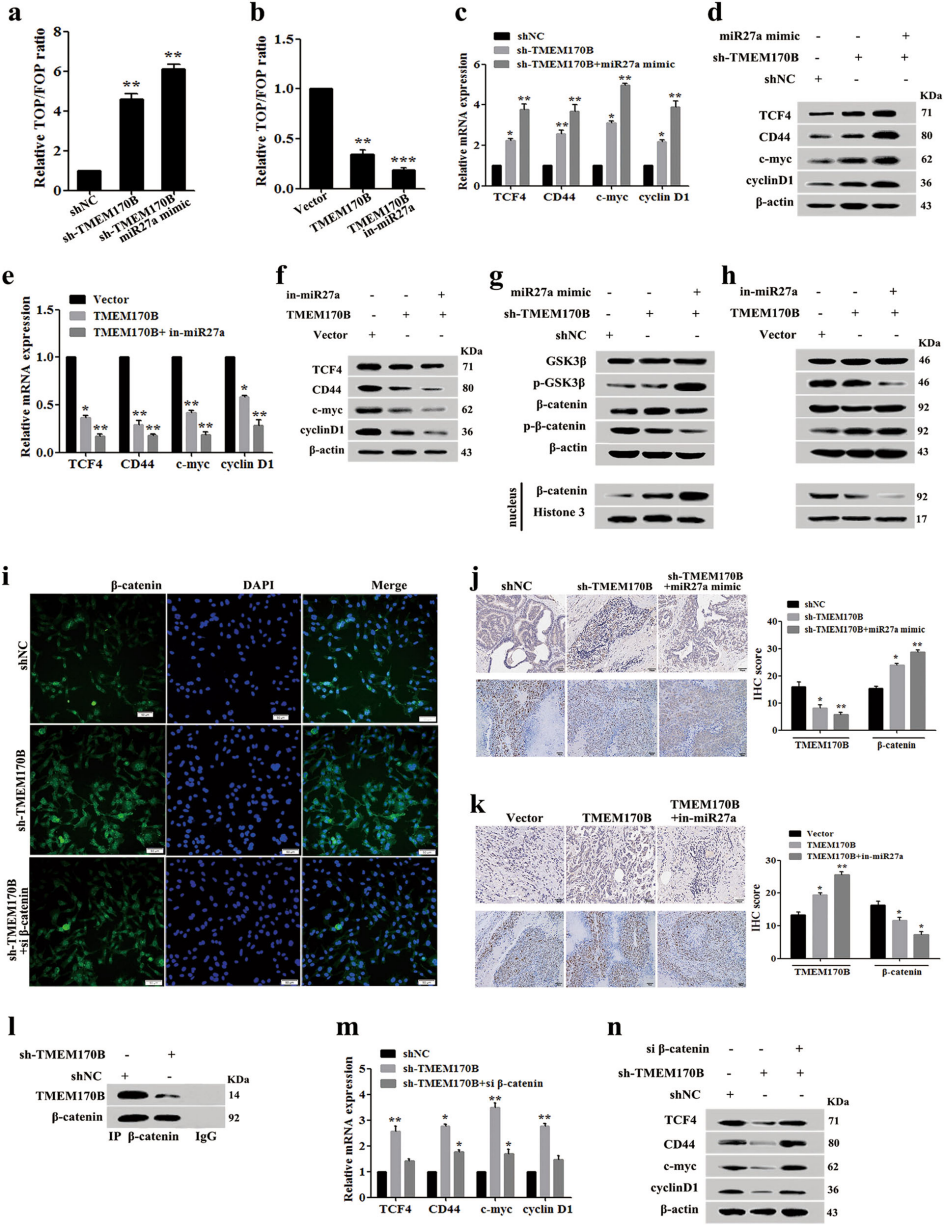
TMEM170B performed as an endogenous inhibitor of the Wnt/β-catenin pathway in breast cancer The Wnt/TCF activity in the stable MCF7 (**a**) or MDA-MB-231 (**b**) cells. The mRNA (**c**) and protein (**d**) levels of β-catenin downstream targets in the stable MCF7 cells were measured by real-time PCR and immunoblotting. The mRNA (**e**) and protein (**f**) levels of β-catenin downstream targets in stable MDA-MB-231 cells were measured by real-time PCR and immunoblotting. Western blot analysis of the protein levels of cytoplasm or nuclear β-catenin in the stable MCF7 (**g**) or MDA-MB-231 (**h**) clones. **i** Immunofluorescence analysis of the subcellular localization of β-catenin in indicated breast cancer cell clones. Representative IHC for TMEM170B and β-catenin expression of the indicated xenograft tumor tissues (*n* = 5) in the stable MCF7 (**j**) or MDA-MB-231 (**k**) clones. Scale bar represents 50 µm. The interaction (**l**) of TMEM170B and β-catenin was analyzed by co-immunoprecipitation. The mRNA (**m**) and protein (**n**) levels of β-catenin downstream targets in the stable MCF7 cells treated with the indicated si β-catenin were detected by real-time PCR and immunoblotting. Data are presented as mean ± S.E.M. *P < 0.05 vs. CTRL, **P < 0.01 vs. CTRL, ***P < 0.001 vs. CTRL

We measured Wnt signaling in breast cancer cells after stable overexpression or knockdown of TMEM170B, which is a direct target of miR-27a, to investigate the regulation of TMEM170B on the Wnt/β-catenin pathway. We first detected the effect of TMEM170B on the endogenous Wnt/TCF activity in breast cancer. As expected, TMEM170B knockdown significantly increased Wnt/TCF activity in MCF7 cells (Fig. 5a), whereas TMEM170B overexpression dramatically inhibited Wnt/TCF activity in MDA-MB-231 cells (Fig. 5b). We further studied the expression of reported Wnt targets using RT-PCR and immunoblotting. Knockdown of TMEM170B consistently induced the mRNA and protein levels of TCF4, CD44, c-myc, and cyclin D1 in MCF7 cells (Fig. 5c, d). whereas overexpression of TMEM170B inhibited the mRNA and protein levels of TCF4, CD44, c-myc, and cyclin D1 in MDA-MB-231 cells (Fig. 5e, f). Consistent with the functional results, miR-27a had a negative role in the molecular regulation of TMEM170B.

In the canonical Wnt pathway^23^, the Wnt ligand brings together a Frizzled receptor and an LRP co-receptor, which represses β-catenin phosphorylation and increases β-catenin protein stability, allowing it to translocate to the nucleus where it functions as a transcriptional co-activator of the TCF/LEF family. Based on the observed effect of TMEM170B on activated β-catenin levels, we further investigated the effect of TMEM170B on β-catenin nuclear translocation. Analysis of nuclear and cytoplasmic extracts showed that knockdown of TMEM170B markedly inhibited the levels of β-catenin phosphorylation in the cytoplasm and induced its nuclear translocation (Fig. 5g). In contrast, TMEM170B overexpression notably induced the levels of β-catenin phosphorylation in the cytoplasm and inhibited nuclear translocation (Fig. 5h). However, the total β-catenin expression levels were not affected by TMEM170B. Since β-catenin phosphorylation requires GSK3β, we studied the GSK3β activities following the knockdown or overexpression of TMEM170B. Interestingly, neither TMEM170B nor miR-27a altered total GSK3β expression, but the levels of GSK3β phosphorylation was modulated by miR-27a (Fig. 5g, h), indicating that TMEM170B may directly regulate β-catenin expression independently of GSK-3β. The negative regulation of β-catenin nuclear translocation by TMEM170B was also confirmed by immune-fluorescence (Fig. 5i). Additionally, the shTMEM170B MCF7 xenografts displayed higher expression levels of nuclear β-catenin (Fig. 5j), whereas xenografts overexpressing TMEM170B MDA-MB-231 had lower expression levels (Fig. 5k).

To further address the regulatory mechanism of TMEM170B, we performed endogenous IP assays using an anti-β-catenin antibody. Interaction between endogenous TMEM170B and β-catenin was detected in MCF7 cells, and knockdown of TMEM170B decreased these interaction levels (Fig. 5l). In addition, MCF7 cells with stable TMEM170B knockdown were transfected with si β-catenin to explore the role of β-catenin in TMEM170B-mediated breast cancer progression. RT-PCR and immunoblotting assays showed that silencing of β-catenin could significantly reduce Wnt/TCF activity, antagonizing the effects of the TMEM170B knockdown (Fig. 5m and n). These results showed that TMEM170B suppressed breast cancer progression by acting as an endogenous inhibitor of β-catenin destabilization in the Wnt/β-catenin pathway.

### TMEM170B is a clinical prognostic hallmark for breast cancer patients

**Fig. 6:**
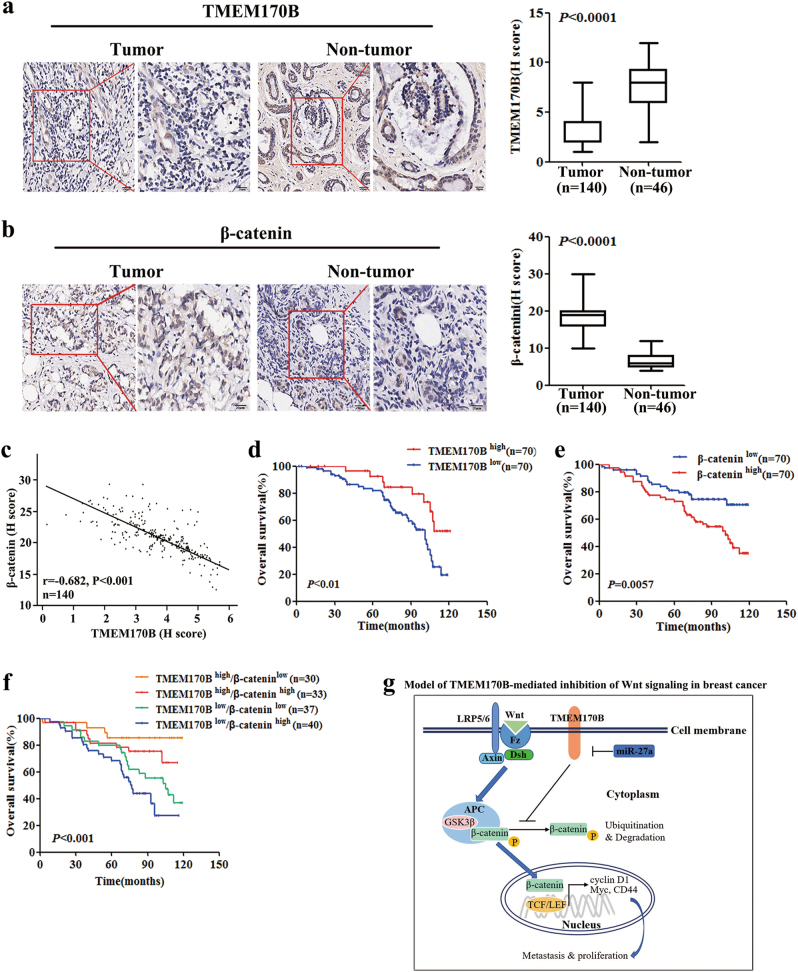
The clinical prognostic value of TMEM170B in breast cancer TMEM170B (**a**) or β-catenin (**b**) levels in clinical breast cancer tissues and noncancer tissues. Scale bar represents 50 µm (left) or 20 µm (right). The correlation of (**c**) TMEM170B and β-catenin expression in clinical breast cancer tissues. **d**,** e** Kaplan–Meier analysis of the overall survival in the clinical BRCA patients with different TMEM170B (**d**), β-catenin (**e**) expression, or TMEM170B/β-catenin expression patterns (**f**). A proposed model (**g**) of TMEM170B functions in the Wnt pathway in breast cancer progression. TMEM170B inhibits β-catenin stabilization and nucleus translocation, which reduces the activity of Wnt targets and inhibits the breast cancer proliferation and metastasis. Values are shown as mean ± S.E.M. *P < 0.05 vs. CTRL, **P < 0.01 vs. CTRL, ***P < 0.001 vs. CTRL

To determine whether TMEM170B expression levels are related to the clinical breast cancer progression, we analyzed the association between TMEM170B and the clinicopathological status of breast cancer patients in the TCGA cohort (Supplementary Tables 1 and 2) and cohort 2 (Supplementary Table 3). As shown in TCGA cohort, statistical analysis represented a strong correlation between miR-27a expression and TMEM170B levels with clinical M stage (*P* = 0.007), ER status (*P* < 0.001), PR status (*P* = 0.001), and Her2 status (*P* < 0.001). In cohort 2 with 140 clinical BRCA patients and 46 normal breast tissues, the TMEM170B expression and β-catenin levels were examined using IHC assay. We found that TMEM170B expression was more abundant in the normal tissues (Fig. 6a), while β-catenin expression occurred mainly in breast cancer tissues (Fig. 6b). In addition, TMEM170B expression was inversely correlated with the β-catenin levels (*r* = −0.628, *P* < 0.001, Fig. 6c). Kaplan–Meier analysis revealed that the BRCA patients with a lower TMEM170B expression had a shorter OS time (19% vs. 52%, *P* < 0.01, Fig. 6d), whereas the patients with higher β-catenin expression were significantly correlated with a reduction in OS time (33% vs. 70%, *P* < 0.01, Fig. 6e). Moreover, the OS of the BRCA patients with TMEM170B-low/β-catenin-high levels were shorter than the patients in other three groups (24%, *P* < 0.001, Fig. 6f). Correlation regression analysis showed that the downregulation of TMEM170B was significantly correlated with the pathological stage (*P* = 0.035) and the TNM (tumor, node and metastasis) status (T status, *P* = 0.028), and upregulated β-catenin expression was significantly correlated with the pathological stage (*P* = 0.014, Supplementary Table 3). Univariate and multivariate Cox regression analysis revealed that the pathological stage, TMEM170B and β-catenin expression were independent prognostic factors for OS in the breast cancer patients (Supplementary Table 4). In addition, significantly lower TMEM170B levels have been found in some other types of human cancer (Supplementary Fig. 5a) compared to adjacent normal tissues (TCGA database). Interestingly, the lower expression of TMEM170B significantly decreased the OS ratio in human uterine (37% vs. 68%, *P* < 0.05, Supplementary Fig. 5b) and renal cell carcinoma (69% vs. 88%, *P* < 0.05, Supplementary Fig. 5c). Our data strongly suggested that an elevated TMEM170B expression inhibited breast cancer progression and may be a clinical prognostic target in multiple malignant tumors.

## Discussion

Despite improvements in the treatment of breast cancer, novel markers and therapeutic targets are urgently needed for the early diagnosis, and prevention of metastasis and for the effective treatment of human breast cancer. In this study, our results revealed several unexpected findings with possible clinical implications. Importantly, we searched publicly recognized^24–26^ high-throughput RNA sequencing databases for cancer prognosis, diagnosis, and therapy. Based on the combination of various bioinformatics analyses and dual-luciferase assays, we unexpectedly discovered that TMEM170B was a new direct target of miR-27a. The in vitro and in vivo results in the present study indicate that TMEM170B may be an endogenous tumor suppressor of BRCA, and play a critical role in multiple tumorigenesis processes, including proliferation, migration, and metastasis. However, other powerful in vivo models, such as dynamic live imaging with luciferase signal, which can track the metastatic colonization in real time would help better explain the importance of TMEM170B in tumor metastasis in vivo.

TMEM170B is a transmembrane protein that belongs to the TMEM170 family, and its important paralog is TMEM170A. A recent study showed that TMEM170A is a new regulator of ER (endoplasmic reticulum) and nuclear envelope morphogenesis^27^. However, the function and molecular mechanism of TMEM170B has not been elucidated. Due to previous publications on the relationship between miR-27a and Wnt signaling, it is known that aberrant Wnt/β-catenin pathway activation initiates transcriptional activation of proteins that are responsible for tumor cell proliferation and metastasis^28^. Our study disclosed a novel mechanistic link between TMEM170B and Wnt/TCF activity through its specific interaction with β-catenin, which results in β-catenin phosphorylation. Notably, β-catenin acts as a transcription co-activator that accelerates target genes, including TCF4, CD44, c-myc, and cyclin D1. Interestingly, miR-27a, and not TMEM170B altered the phosphorylation state of GSK3β, indicating that miR-27a may involve other cross-talk points to regulate the Wnt/β-catenin pathway. Thus, our results identified TMEM170B as a novel regulator that links β-catenin nuclear translocation to the suppression of BRCA progression and metastasis (Fig. 6g). In addition, the Wnt/β-catenin pathway is also critical for many other developmental events, including cell differentiation, stem cell self-renewal, and cytoskeletal rearrangement. Thus, in future studies, it will be interesting to investigate other (β-catenin-dependent) cellular processes and mechanisms by which TMEM170B exerts its suppressive effects on cancer.

Currently, triple-negative breast cancer (TNBC) has higher rates of distant recurrence and worse prognosis compared to other subtypes of breast cancer. The treatment of TNBC patients has been challenging due to the absence of well-defined molecular targets^29^. Our analysis showed that miR-27a expression in TNBC patients was higher than that in non-TNBC patients (Supplementary Fig. 5d), whereas TMEM170B levels in breast cancer patients with TNBC were lower than that in non-TNBC patients (Supplementary Fig. 5e). Thus, these results suggested that TMEM170B could be a signature of TNBC. Future research will be needed to clarify whether TMEM170B can be a therapeutic target in TNBC patients.

In conclusion, our results revealed that TMEM170B, as a functional target of miR-27a, is important for balancing the deregulation of Wnt/β-catenin signaling in breast cancer. This study also identified that high expression of TMEM170B was negatively correlated with the poor prognosis of breast, uterine, and renal cell carcinoma patients. Thus, TMEM170B could be regarded as a novel target in cancer progression. Future research efforts to design novel drugs to activate TMEM170B will provide a new therapeutic strategy to improve breast cancer treatment.

## Materials and methods

### Cell culture and treatment

The breast cancer cell lines MDA-MB-231, MDA-MB-435, BT474, and MCF7 were purchased from American Type Culture Collection (ATCC, Manassas, USA) and cultured in DMEM medium (Gibco, Grand Island, NY). The non-tumorigenic epithelial cell line MCF-10A was cultured in RPMI-1640 medium (Gibco). The growth media were supplemented with 10% fetal bovine serum (Gibco) and 1% penicillin/streptomycin (Invitrogen, USA). Cells were passaged continuously for no more than 6 months after receipt for relevant studies reported herein.

### RNA interference and transfection

The following target siRNA sequences were used in this study: Si β-catenin-1, 5′-GUUAUGGUCCAUCAGCUUU-3′ and Si β-catenin-2, 5′-GCAGUUGUAAACUUGAUUATT-3′. All siRNAs, miRNA-27a mimic and inhibitor (GenePharma Co. Ltd., Shanghai) were transfected into breast cancer cells using Lipofectamine RNAiMAX reagent (Invitrogen). After 6 h, fresh medium was replaced and the cells were grown for 24 h before further assays.

### Vectors construction and cell transfection

Two short hairpin RNA oligonucleotides targeting TMEM170B and negative control were individually cloned into piLenti-siRNA-GFP-Puro lentiviral vectors (abmGood Inc. Canada). Full length human TMEM170B complementary DNA (cDNA) was cloned into pLVX-CMV-GFP-Puro lentiviral vectors (abmGood Inc. Canada). 293T cells were transfected using lipofectamine 3000 (ThermoFisher Scientific) with the necessary lentiviral vector mentioned earlier and packaging vectors pMD2.G (VSV-G protein, addgene), psPAX2 (Gag/Pol protein, addgene). The lentiviral particles were collected and transfected into breast cancer cells as described previously^30^. All the transfected breast cancer cells were selected with puromycin dihydrochloride (Sigma-Aldrich, St. Louis, MO) for 4 weeks. The stable clones were identified using quantitative real-time PCR (qRT-PCR) assay and used for further analysis.

### RNA extraction and qRT-PCR

Total RNA from all cells were isolated using Trizol reagent (Invitrogen, USA) and the first-strand cDNA was generated using the PrimeScript™ RT reagent Kit with genomic DNA Eraser (Takara, China). For miRNA analysis, mature miRNAs were reversely transcribed with Universal cDNA Synthesis Kit (Exiqon, Denmark). qRT-PCR was performed in the StepOne™ real-time pcr system (Bio-Rad CFX96) using SYBR® Green (Takara) and the gene-specific primers shown in Supplementary Table 1. Relative expression was evaluated by a comparative CT method. The mRNA and miRNA levels were normalized against β-actin or U6 small RNA.

### Immunoblotting analysis

All cells for protein extraction were lysed on ice in RIPA buffer (Cell Signaling Technology, Boston, MA). Quantified protein lysates were separated on 8–15% gradient gel and then electro-transferred to polyvinylidene fluoride (Millipore, Bedford, MA) membranes. The membranes were blocked with 5% BSA for 2 h and then incubated with primary antibodies (listed in Supplementary Table 6) overnight at 4 °C. After washing, the membranes were incubated with secondary HRP-conjugated antibodies for 1 h at room temperature. The bands were detected using ECL (enhanced chemiluminescence) kit (Tanon, China) and analyzed with ImageJ software. Relative expression levels of the indicated proteins were compared with β-actin expression.

### Immunostaining assays

For immunostaining, cells were fixed with 4% PFA for 20 min, and permeabilized in 0.1% Triton X-100 (HFH10, ThermoFisher) for 15 min at room temperature. After blocking with 5% BSA for 60 min at room temperature, cells were incubated with the indicated primary antibodies (listed in Supplementary Table 6) overnight at 4 °C. After three washes with PBS, cells were stained with fluorescently labeled secondary antibodies for 1 h at room temperature. Coverslips were mounted with DAPI (Sigma) and examined under a confocal microscope (LSM 800, Zeiss) with a 63×/1.40 oil-immersion objective lens (Plan-Apochromatlan, Zeiss).

### Subcellular protein fractionation

Subcellular fractionation was performed following the protocol from ThermoFisher (https://www.thermofisher.com/order/catalog/product Subcellular Protein Fractionation Kit for Cultured Cells.pdf). In brief, cells were lysed with a subcellular fractionation buffer and cell lysates were centrifuged. The pellet was washed in fractionation buffer and resuspended in cytoplasmic extraction buffer; this is the cytoplasmic fraction. The supernatant was then re-centrifuged at a higher speed (16,000 × *g*), the supernatant, after ultracentrifugation being the membrane fraction. The pellet was resuspended in nuclear extraction buffer giving the nuclear fraction.

### Immunoprecipitation analysis

MCF7 cell with different plasmids extracts were immunoprecipitated as previously described^31^, using β-catenin or non-specific immunoglobulin G (IgG) (Santa Cruz Biotechnology) and protein G-agarose beads (ThermoFisher Scientific) at 4 °C overnight. After centrifugation to pellet the agarose beads, supernatants were subjected to SDS-PAGE and IB (immunoblotting).

### Cell proliferation assays

MDA-MB-231 and MCF7 cells (2 × 10^3^ per well) were seeded in 96-well plates and analyzed using (3-(4,5-dimethylthiazol-2-yl)−2,5-diphenyltetrazolium bromide (MTT, Sigma)) at 24, 48, and 72 h, respectively. Cells viability was measured using a microplate reader (ThermoFisher, USA) at 570 nm.

### Colony-formation assays

MDA-MB-231 and MCF7 cells (500 per well) were seeded in a six-well plate and fresh media replaced every 3 days. After 2 weeks of culture, cells were fixed by ice-cold methanol and stained with 1% crystal violet. Colonies were counted directly on the plate and statistical significance was calculated based on four independent experiments.

### Cell migration and invasion assays

MDA-MB-231 and MCF7 cells under various experimental conditions were resuspended in serum-free media. A total of 5 × 10^4^ cells per well were added to the upper compartment of the transwell chamber containing 8-μm pores with (invasion assay) or without (migration assay) matrigel. The chamber was placed into a 24-well plate with 600 μl of DMEM containing 10% FBS and incubated for 24 h. After removal of non-invaded or non-migrated cells, cells that had invaded/migrated through the filter were fixed with ethanol, stained with crystal violet solution, and counted with a digital microscope. (CKX41, Olympus, Japan).

### Wound-healing assay

MDA-MB-231 and MCF7 cells (1 × 10^4^ per well) were seeded in a 96-well plate and incubated for 24 h. A straight wound line was made by scraping with a sterile 20-μl pipette tip across the cell monolayer. Cells were washed with PBS and cultured in DMEM supplemented with 1% FBS for 24 and 48 h. The movement of cells toward the wound at different time points were captured under the microscope (×10 magnification).

### Bioinformatics analysis

GEO data retrieval: three miRNA expression profiling data sets (GSE 26659/68085/40525) of breast cancer were used^32,33^ from GEO Data portal (https://www.ncbi.nlm.nih.gov/geo/).

The Cancer Genome Atlas data retrieval^34^: the gene expression data (including miRNA and RNA expression data) of breast cancer and corresponding clinical data were downloaded from TCGA data set (https://tcga-data.nci.nih.gov/). miRNA-seq and mRNA-seq data of 1071 clinical BRCA samples and 104 non-tumor tissue samples were downloaded from TCGA data sets. Log2-normalized counts were performed to describe the expression of mRNAs, and “reads per million miRNA” were used to determine the miRNAs expression.

### miRNA target predictions

The potential targets of miR-27a were obtained from miRDB data sets (http://www.mirdb.org/miRDB/). Method 1 used Starbase 2.0 (http://starbase.sysu.edu.cn/) to obtain the decreased genes of predicted top100 targets of miR-27a in BRCA. Method 2 used three specific programs: Targetscan (www.targetscan.org), Pictar (http://pictar.mdc-berlin.de), and miRanda (http://pictar.mdc-berlin.de) to further predict the targets of miR-27a.

### Dual-luciferase reporter assays

The 3′-UTRs luciferase reporters of the *TMEM170B*,* GPAM*,* PPAP2B*,* ST6GALNAC3*,* EYA1*, and *PPARG* gene were generated by annealing the forward and reverse oligonucleotides of 3′-UTR of each gene, which were then cloned into pmirGLO Vector (Promega, USA). The relative luciferase activity was calculated as the ratio between firefly luciferase activity and Renilla luciferase activity according to the manufacturer’s instructions (Promega).

### Biotinylated RNA pull-down assay

Breast cancer cells pull-down experiments with biotinylated miR-27a or biotinylated TMEM170B (GenePharma, China) were conducted as described previously^35,36^. Briefly, cells are collected and lysed 24 h post transfection with RNAimax (Invitrogen, USA). Complexes of biotinylated RISC and RNA are pulled down with strepatividine coated magnetic beads (Invitrogen). After wash steps, the captured RNA is purified and analyzed by qRT-PCR.

### RNA immunoprecipitation assay

The RIP experiments were performed by using Magna RIP™ RNA-Binding Protein Immunoprecipitation Kit (Millipore, Bedford, MA). Magnetic beads were pre- incubated with an AGO2 antibody (Millipore), and were immunoprecipitated with cells lysates at 4 °C overnight. RNA was purified from RISC complex and then analyzed by qRT-PCR assay.

### TOP/FOP luciferase reporter assay

Transcriptional activity assays were performed using the Luciferase Assay System (Genepharma, China) according to the manufacturer’s instructions^37^. Briefly, cells were transfected with siRNAs targeting β-catenin or the scramble control. Cells were co-transfected with the M50 Super 8× TOP Flash or M51 Super 8× TOP Flash by Lipofectamine 3000 after 24 h post transfection. In some of the experiments, cells were also co-transfected with TMEM170B expression vector and miR-27a mimic or inhibitor. The luciferase activity of each sample was normalized against Renilla luciferase activity to monitor transfection efficiency. Twenty-four hours after transfection of plasmids, cells were lysed and luciferase activity was measured using the dual-luciferase reporter (DLR) assay Kit (Promega). Firefly and Renilla luciferase activities were determined using a luminometer and normalized.

### In vivo tumorigenicity

Female Balb/c nude mice (4–5 weeks; Cavens Lab Animal Co.) were cared for according to the Provisions and General Recommendation of Chinese Experimental Animals Administration Legislation. Mice were accustomed to local conditions for >1 week and maintained under a 12-hour dark and light cycle, with water and food. All animal experiments complied with IACUC (institutional animal care and use committee) regulations. For xenograft assays, 5 × 10^6^ indicated breast cancer cells were orthotopically implanted into the mammary fat pads of nude mice. The mammary primary tumor growth rates were monitored by measuring tumor length (*L*) and width (*W*) with a caliper. Volume was calculated as 0.50 × *W*^2^ × *L*. Five weeks after tumor implantation, all the mice were killed (euthanized). The tumors and major organs were removed, examined, and photographed.

### Hematoxylin and eosin and immunohistochemistry

Tissue samples were fixed in 10% formalin at least for 24 h, embedded in paraffin, sectioned at 5-μm thickness and stained with hematoxylin and eosin (H&E). The histology of different tissues in each group of mice were observed by microscopy (20×, Olympus). The paraffin tumor sections were individually incubated with primary antibodies for TMEM170B or β-catenin, and followed by incubation with the secondary antibodies and detected with DAB kit (Abcam).

### Tissue microarrays

Tissue microarrays containing breast cancer tissues (HBre-Duc140Sur-02) and normal breast tissues (HBre-Duc046Sur-02) were constructed by Shanghai Outdo Biotech^38^. All of the tissues were validated by H&E staining. The TMEM170B and β-catenin levels obtained by immunostaining were scored by multiplying the extent (0–100) and intensity (0–3) for each tissue point.

### Statistics

All data were presented as mean ± S.E.M. Statistical significance was analyzed using GraphPad Prism 6 Software and determined by Student’s *t* test, one-way analysis of variance, Spearman correlation coefficients, and log-rank test. *P* values <0.05 were considered statistical significance.

## Electronic supplementary material

Supplementary Figure 1

Supplementary Figure 2

Supplementary Figure 3

Supplementary Figure 4

Supplementary Figure 5

Supplementary information
