# Supplementary figures and images for: Transmembrane protein 170B is a novel breast tumorigenesis suppressor gene that inhibits the Wnt/β-catenin pathway
Source: Cell Death Dis. 2018 Jan 24;9(2):91. doi: 10.1038/s41419-017-0128-y (PMC5833782; doi:10.1038/s41419-017-0128-y)

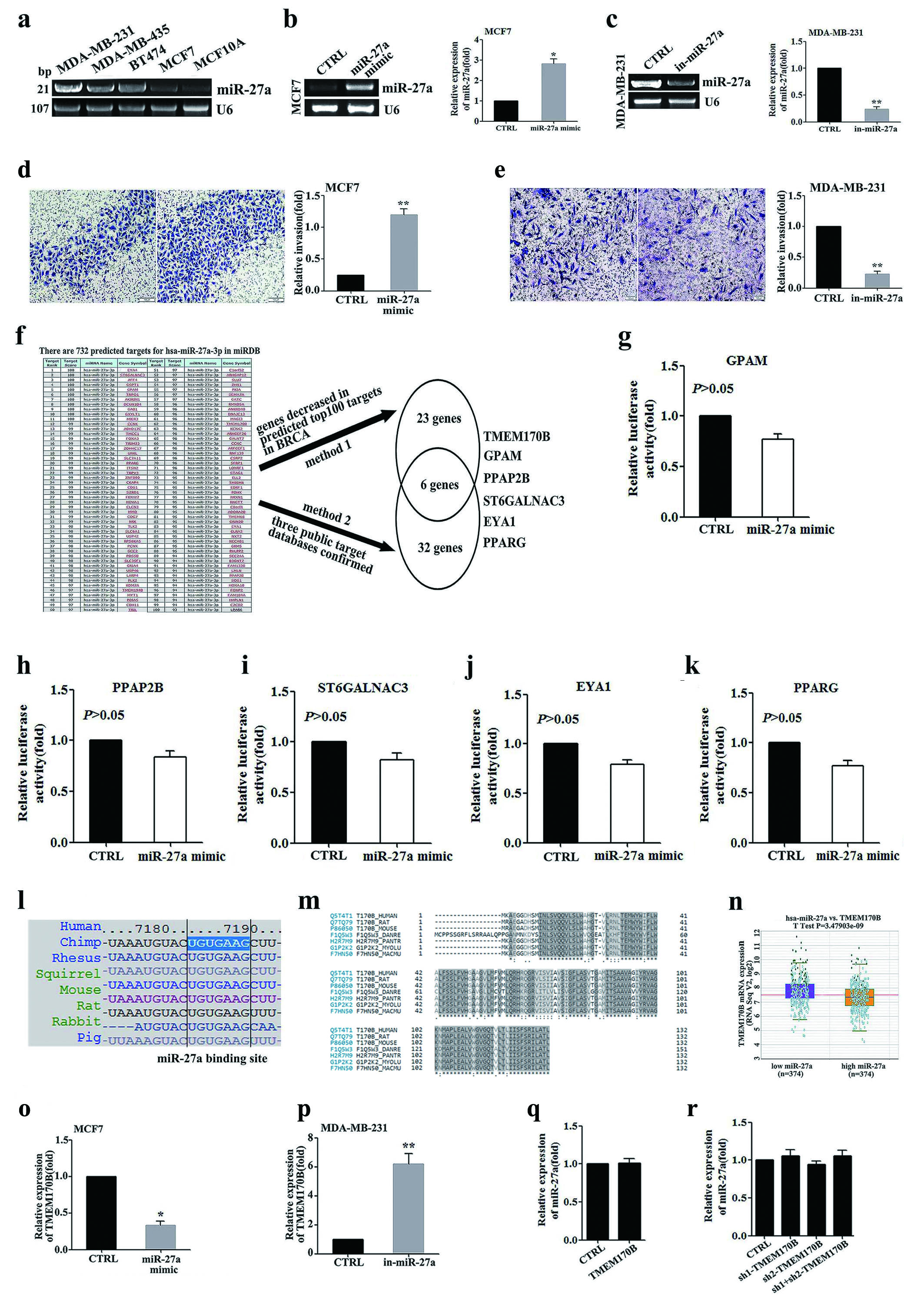

Supplement: Supplementary file 1 — Supplementary Figure 1 [file 41419_2017_128_MOESM1_ESM.jpg]

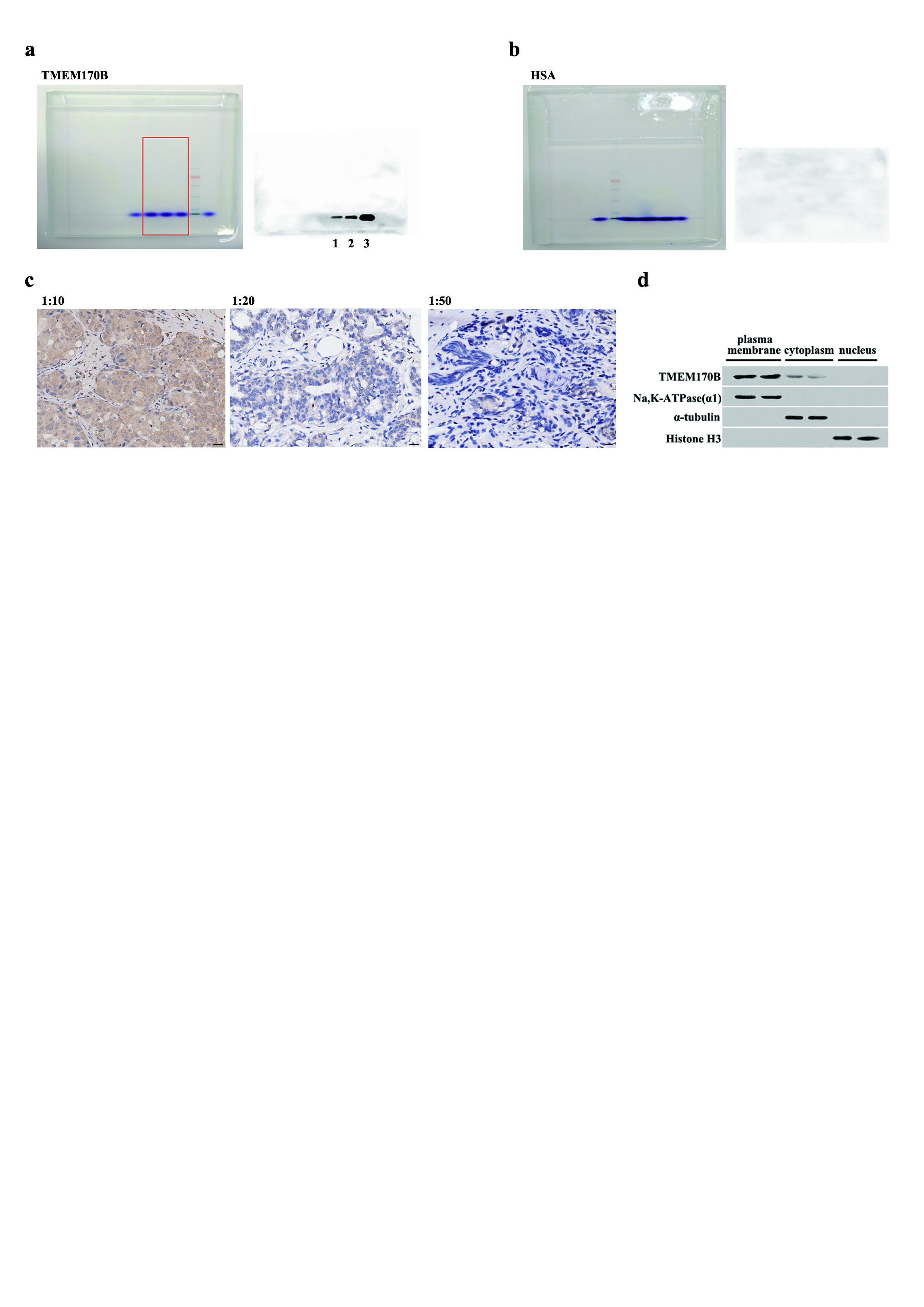

Supplement: Supplementary file 2 — Supplementary Figure 2 [file 41419_2017_128_MOESM2_ESM.jpg]

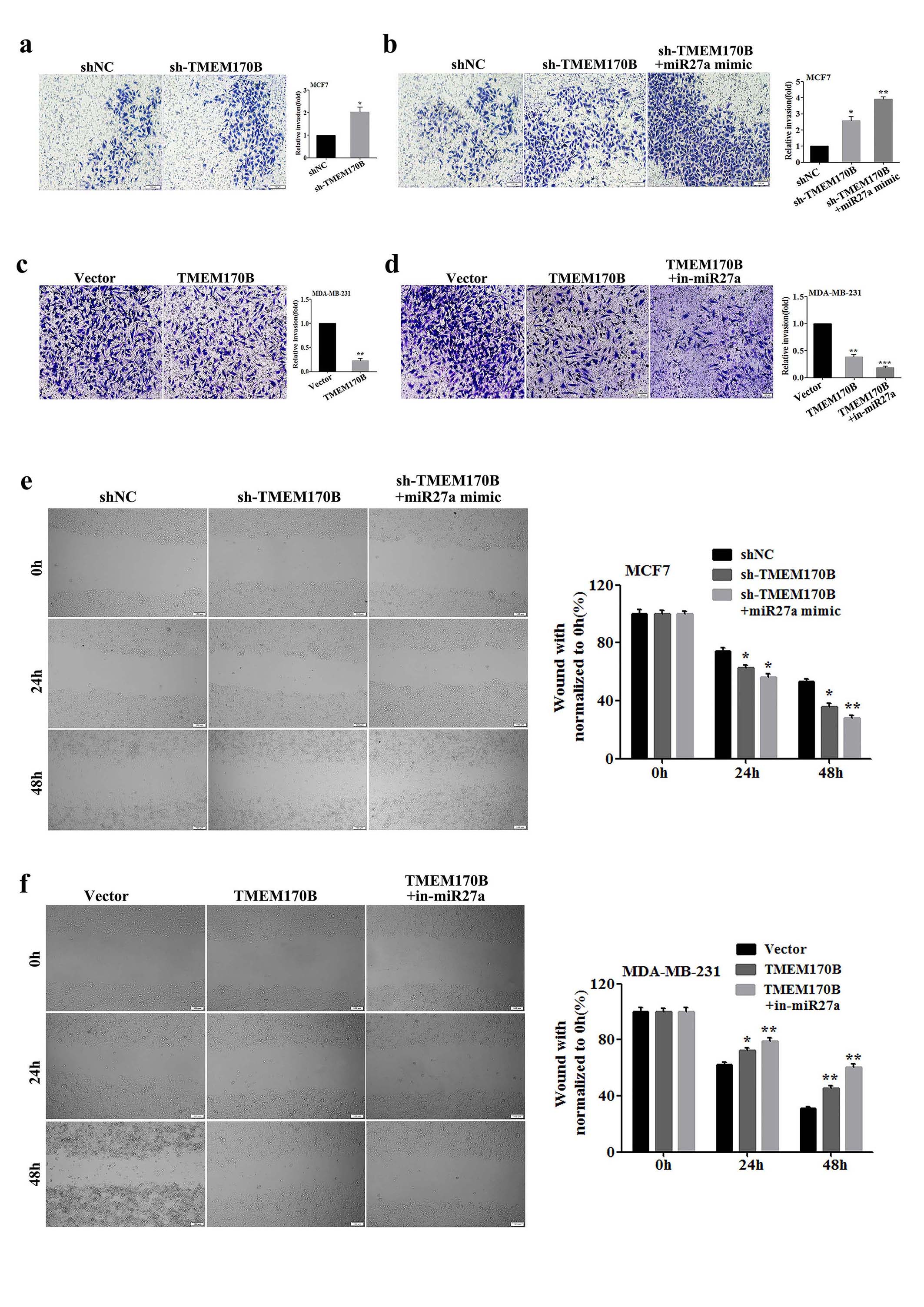

Supplement: Supplementary file 3 — Supplementary Figure 3 [file 41419_2017_128_MOESM3_ESM.jpg]

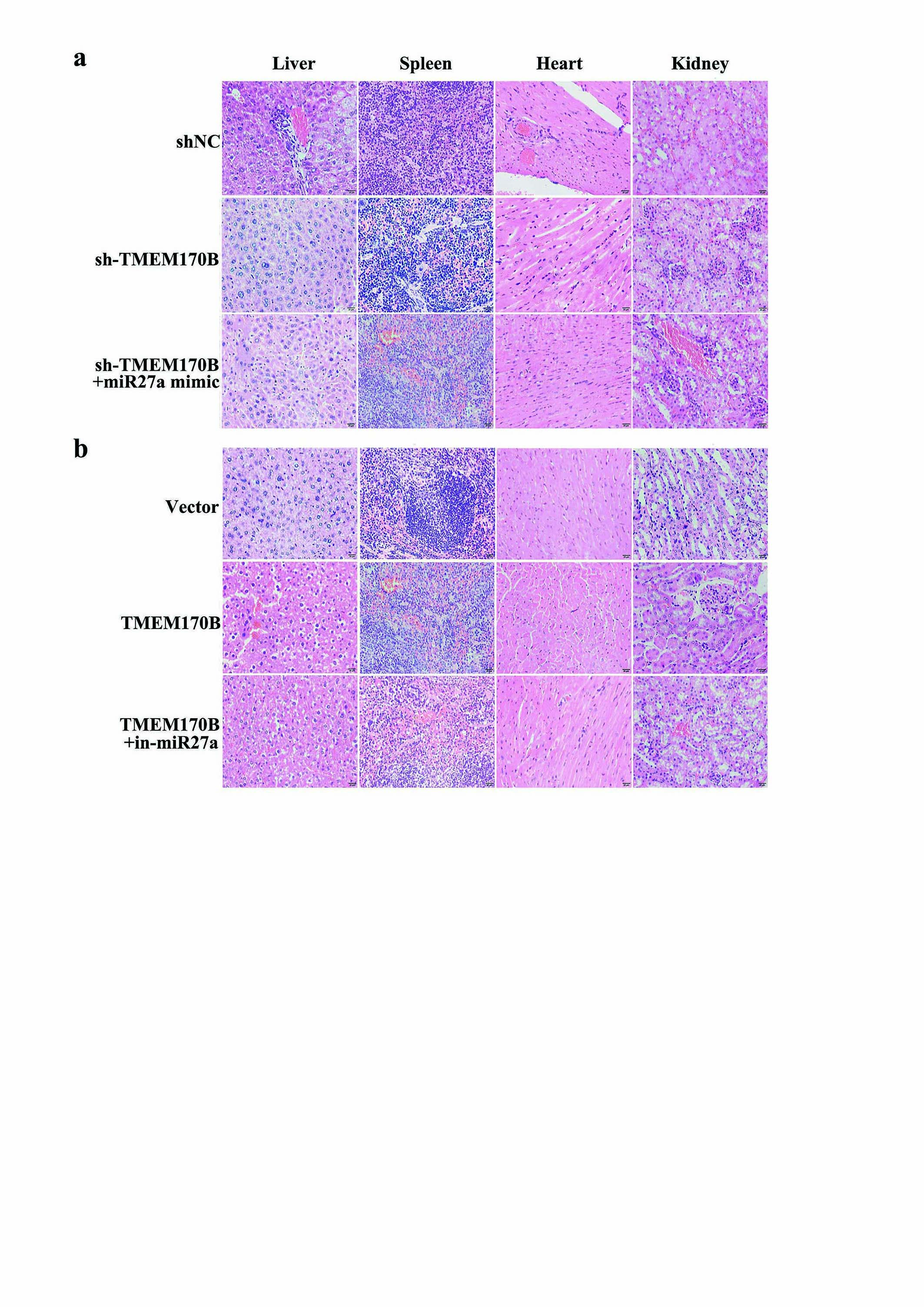

Supplement: Supplementary file 4 — Supplementary Figure 4 [file 41419_2017_128_MOESM4_ESM.jpg]

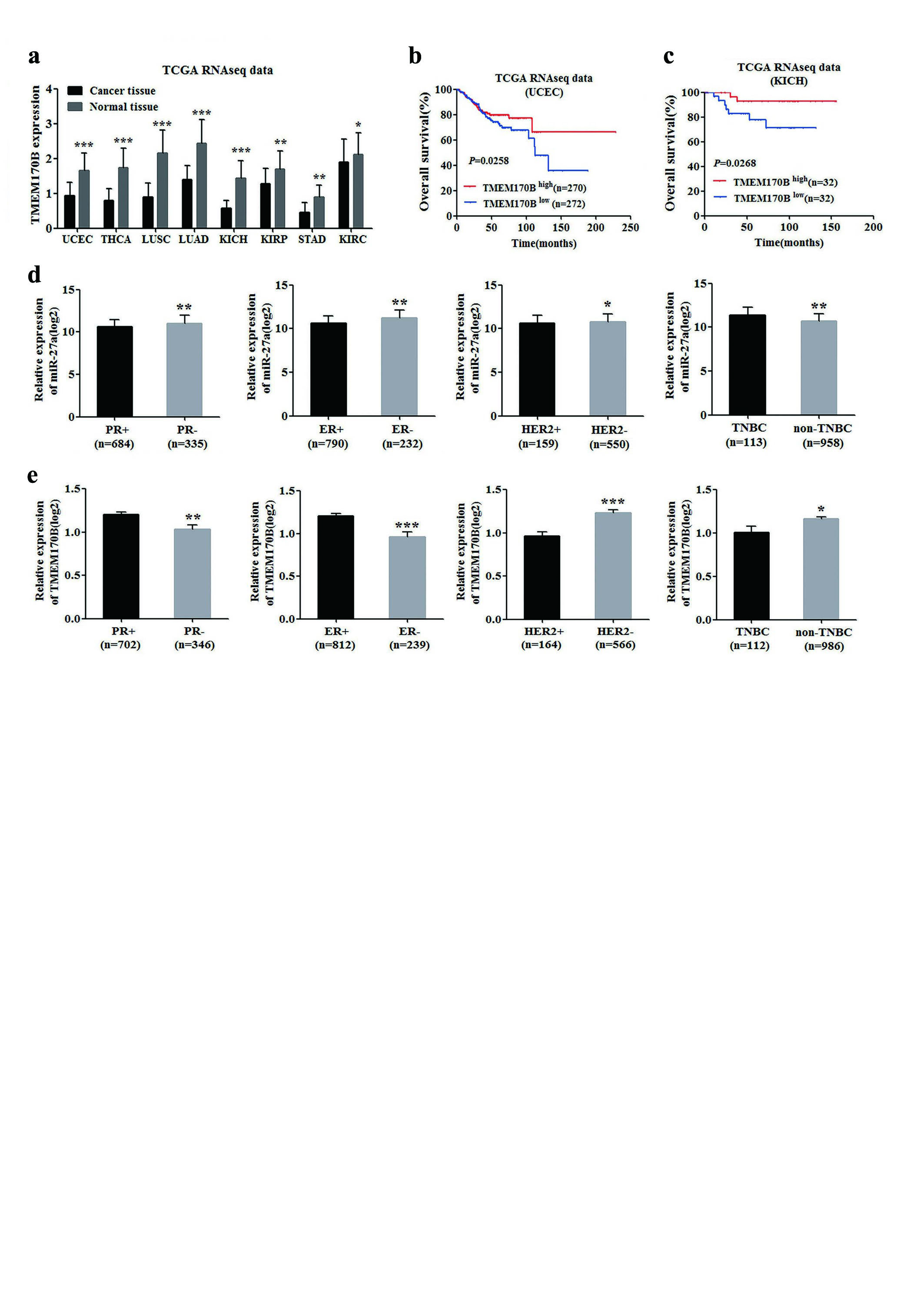

Supplement: Supplementary file 5 — Supplementary Figure 5 [file 41419_2017_128_MOESM5_ESM.jpg]
