# Supplementary material for: Transmembrane protein 170B is a novel breast tumorigenesis suppressor gene that inhibits the Wnt/β-catenin pathway
Source: Cell Death Dis. 2018 Jan 24;9(2):91. doi: 10.1038/s41419-017-0128-y (PMC5833782; doi:10.1038/s41419-017-0128-y)
Supplement: Supplementary file 6 — Supplementary information [file 41419_2017_128_MOESM6_ESM.pdf]

Supplementary information

Supplementary Figure 1 The effect of miR-27a on breast cancer invasion and relationship between miR-27a and TMEM170B. **(a)** The miR-27a expression in breast cancer cell lines. **(b** and **c)** miR-27a levels in MDA-MB-231 cells transfected with miR-27a inhibitor **(b)** or in MCF7 cells transfected with miR-27a mimic **(c)**. **(d** and **e)** Cell invasion assay was performed on MDA-MB-231 cells with miR-27a inhibitor **(d)** or MCF7 cells with miR-27a mimic **(e)**. **(f**) Venn with the overlap of potential genes targeted by miR-27a, as analyzed in the TCGA database and predicted by the following algorithms: TargetScan, miRanda, and PicTar. **(g-k)** The 3'-UTR luciferase reporter activities of GPAM, PPAP2B, ST6GALNAC3, EYA1 or PPARG gene transfected with miR-27a mimic or control. **(l)** Schematic depiction of the sequence of the miR-27a binding site within the 3'UTR of TMEM170B in different species. **(m)** Multiple sequence alignment of TMEM170B homologues in representative species from UniProtKB/Swiss-Prot database. Fully conserved amino acids are highlighted in dark gray and indicated by a star (∗) below, amino acids with strongly similar properties are highlighted in light gray and indicated by a colon (:) and those with weakly similar properties are in white and indicated by a period (.). **(n)** The relation of TMEM170B expression with miR-27a levels in the Starbase2.0 database. **(o** and **p)** TMEM170B expression in the MDA-MB-231 cells transfected with miR-27a mimic **(o)** or inhibitor **(p)**. **(q** and **r)** The effect of TMEM170B overexpression **(q)** or knockdown **(r)** on miR-27a levels. Data are presented as mean ± S.E.M. n=4, *P<0.05 *versus* CTRL, **P<0.01 *versus* CTRL.

Supplementary Figure 2 The specificity of TMEM170B antibody and subcellular localization of TMEM170B. **(a)** The complete gel (left) and immunoblotting images (right) of MCF7 cells protein with different concentration of TMEM170B antibody. Lane1, 1:1000; lane2, 1:500; lane3, 1:250. **(b)** The complete gel (left) and immunoblotting images (right) of the negative control (human serum albumin, HAS) with TMEM170B antibody. **(c)** Representative image of TMEM170B expression with different concentrations antibody by IHC assay. Scale bar represents 20 μm. **(d)** Immunoblot analysis of the subcellular fractionation of TMEM170B. Na,K-ATPase (α 1) was used as a marker for normalization of the plasma membrane fraction; α-tubulin was used as a marker for

normalization of the cytoplasmic fraction. Histone3 (H3) was used as a marker for normalization of the nuclear fraction.

Supplementary Figure 3 The effect of TMEM170B on breast cancer cell migration and invasion. **(a** and **b)** Cell invasion assay of indicated MCF7 cells without **(a)** or with **(b)** miR-27a mimic. **(c** and **d)** Cell invasion assay of indicated MDA-MB-231 cells without **(c)** or with **(d)** miR-27a inhibitor. **(e)** Cell wound-healing ability was measured for MCF7 cells with TMEM170B knockdown without or with miR-27a mimic. **(f)** Cell wound-healing ability was measured for MDA-MB-231 cells with overexpressing TMEM170B without or with miR-27a inhibitor. Scale bar represents 100 μm. Values are shown as mean ± S.E.M. n=4, *P<0.05 *versus* CTRL, **P<0.01 *versus* CTRL, ***P<0.01 *versus* CTRL.

Supplementary Figure 4 Hematoxylin-eosin staining of transplanted tumor xenografts from each treatment group. **(a)** Representative images of paraffin embedded liver, spleen, heart and kidney sections of tumor xenografts model in MCF7 cells with TMEM170B shRNA without or with miR-27a mimic (n=5). **(b)** Representative images of paraffin embedded liver, spleen, heart and kidney sections of tumor xenografts model in MDA-MB-231 cells with overexpressing TMEM170B without or with miR-27a inhibitor (n=5). Scale bar represents 50 μm.

Supplementary Figure 5 The clinical prognostic value of TMEM170B in different cancer. **(a)** TMEM170B levels of different cancer types in the TCGA database. UCEC, uterine corpus endometrial carcinoma; THCA, thyroid carcinoma; LUSC, lung squamous cell carcinoma; LUAD, lung adenocarcinoma; KICH, kidney renal chromophobe cell carcinoma; KIRP, kidney renal papillary cell carcinoma; STAD, stomach adenocarcinoma; KIRC, kidney renal clear cell carcinoma. **(b** and **c)** Overall survival of UCEC **(b)** or KICH **(c)** patients with different TMEM170B levels is represented as a Kaplan-Meier plot. **(d)** miR-27a expression in patients of different breast cancer subtypes based on miRNA sequencing (FPKM) data from TCGA. **(e)** TMEM170B expression in patients of different breast cancer subtypes based on RNA sequencing (RNA-seq) data from TCGA database. Log2-normalized read count was shown. Data show mean ± S.E.M. *P<0.05, **P<0.01, ***P<0.01.

Supplementary Table 1

The association of miR-27a and TMEM170B levels with clinic pathological characteristics of BRCA patients (n=1071)

	miR-27a			TMEM170B			β-catenin		
Variables	Low	High		Low	High		Low	High	
	n=535	*n*=536	*P*	*n*=535	*n*=536	*P*	*n*=535	*n*=536	*P*
Gender									
Male	8	4		7	5		5	7	
Female	527	532	0.472	528	531	0.685	530	529	0.519
Age(Years)									
< 60(568)	266	302		286	282		301	267	
≥ 60(502)	268	234	**0.019**	249	253	0.128	263	239	**0.037**
Pathologic stage									
I+II	393	404		398	399		410	387	
III	142	132	0.354	137	137	0.615	135	139	0.428
T status									
T1+2	451	443		447	447		444	450	
T3+4	84	93	0.583	88	89	0.709	86	91	0.138
N status									
N0+1(859)	427	432		426	433		430	429	
N2+3(212)	108	104	0.581	109	103	0.451	106	106	**0.025**
M status									
M0(889)	438	451		428	461		430	459	
M1(182)	97	85	**0.025**	107	75	**0.007**	95	87	**0.016**
Lymph node count									
≤ 9	234	252		245	241		240	246	

> 9	239	227	0.478	237	229	0.301	235	231	0.375
ER									
Positive (790)	446	344		374	416		432	358	
Negative (232)	68	163	**<0.001**	144	87	**<0.001**	97	135	**0.005**
PR									
Positive (684)	386	298		319	365		365	319	
Negative (335)	125	210	**<0.001**	200	135	**0.001**	187	148	0.185
Her2									
Positive (159)	83	76		100	59		91	68	
Negative (550)	270	280	**0.032**	259	291	**<0.001**	265	285	**0.017**

NOTE: **P* < 0.05 is significant. All analyses were conducted using $\chi 2$ tests and Fisher's exact tests.

A "low" versus "high" expression levels were defined according to their cut-off values, which were defined as the median value of the whole cohort of tested patients.

Supplementary Table 2

Univariate and Multivariate Cox Regression Analyses of potential factors for survival in BRCA patients (n=1071)

Variables	Univariate analysis		Multivariate analysis	
	HR(95%CI)	P-value	HR(95%CI)	P-value
Gender, male vs female	0.669 (0.418-1.271)	0.285		n.s.
Age(Years), < 60 vs ≥ 60	0.927 (0.812-1.325)	0.351		n.s.
Pathologic stage, I+II vs III	1.247 (1.023-1.899)	**0.028**		n.s.
T status, T1+2 vs T3+4	1.854 (1.328-2.105)	0.059		n.s.
N status, N0+1 vs N2+3	1.753 (1.487-2.789)	0.124		n.s.
M status, M0 vs M1	2.532 (1.523-3.651)	0.318		n.s.
ER status, positive vs negative	2.254 (2.031-3.128)	0.072		n.s.
PR status, positive vs negative	1.893 (1.429-2.316)	0.131		n.s.
HER2 status, positive vs negative	2.310 (1.326-3.184)	0.205		n.s.
High miR-27a vs low miR-27a	2.378 (1.429-2.953)	**<0.001**	2.934 (1.249-4.214)	**0.000**
High TMEM170B vs low TMEM170B	2.019 (1.138-3.028)	**<0.001**	2.456 (1.578-3.913)	**0.016**
High β-catenin vs low β-catenin	2.161 (1.237-3.137)	**0.015**	2.012(1.014-2.865)	**0.005**

NOTE: n.s. is not significant; **P* < 0.05 is significant.

Supplementary Table 3

The association of TMEM170B and β-catenin levels with clinic pathological characteristics of BRCA patients(n=140)

Variables	TMEM170B			β-catenin		
	Low	High		Low	High	
	n=73	*n*=67	*P*	*n*=68	*n*=72	*P*
Gender						
Male	0	0		0	0	
Female	73	67	0.351	68	72	0.429
Age(Years)						
< 60	31	24		27	28	
≥ 60	42	43	0.492	39	46	0.268
Pathologic stage						
I+II	30	33		38	33	
III	40	39	**0.035**	30	47	**0.014**
T status						
T1+2	10	5		7	8	
T3+4	74	51	**0.028**	62	63	0.420
N status						
N0+1	57	54		46	65	
N2+3	18	11	0.419	14	15	0.373
Lymph node count						
≤ 11	44	31		43	32	
> 11	38	27	0.478	37	28	0.301
Tumor size (cm)						
≤ 5	35	37		29	41	
> 5	38	30	0.620	39	31	0.543

Supplementary Table 4

Univariate and Multivariate Cox Regression Analysis of potential factors for survival in BRCA patients (n=140)

Variables	Univariate analysis		Multivariate analysis	
	HR(95%CI)	P-value	HR(95%CI)	P-value
Gender, male vs female	0.534 (0.128-1.102)	0.236		n.s.
Age(Years), < 60 vs ≥ 60	0.210 (0.092-1.358)	0.615		n.s.
Pathologic stage, I+II vs III	2.193 (0.815-3.217)	**0.027**		**0.031**
T status, T1+2 vs T3+4	1.591 (0.629-2.256)	0.129		n.s.
N status, N0+1 vs N2+3	1.264 (0.716-2.318)	0.312		n.s.
Tumor size(cm), ≤ 5 vs >5	1.905 (1.136-2.245)	0.251		n.s.
High TMEM170B vs low TMEM170B	2.419 (1.385-4.103)	**0.000**	2.643 (1.416-4.825)	**0.038**
High β-catenin vs low β-catenin	2.521 (1.205-4.302)	**0.012**	2.015 (1.034-3.926)	**0.003**

Supplementary Table 5

Primers sequence used in the study

Gene name	Forward	Reverse
miR-27a	5´-TTCACAGTGGCTAAGTTCCGC-3´	5´-GTGCAGGGTCCGAGGT-3
U6	5´-CTCGCTTCGGCAGCACA-3´	5´-AACGCTTCACGAATTTGCGT-3´
TMEM170B	5´-GGATCCTCGCTACACTTTGAG-3´	5´-TCCTTATGCTTTGACCTGCTC-3´
β-actin	5´-GGGAAATCGTGCGTGACATTAAG-3´	5´-GTCAGGCAGCTCGTAGCTCT-3´
β-catenin	5´-ACTACCACAGCTCCTTCTCT-3´	5´-AAATCCCTGTTCCCACTCATAC-3´
CD44	5´-GCAGGTATGGGTTCATAGAAGG-3´	5´-GGTGTTGGATGTGAGGATGT-3´
TCF4	5´-CCACCCATTTCTTTGCTGAAC-3´	5´-CCCTGACTCTTAACACCAACTC-3´
Cyclin D1	5´-GGGTTGTGCTACAGATGATAGAG-3´	5´-AGACGCCTCCTTTGTGTTAAT-3´
c-myc	5´-TGAGGAGGAACAAGAAGATG-3´	5´-ATCCAGACTCTGACCTTTT-3´

Supplementary Table 6

Primary antibodies used in this study

Antibodies	Manufacturer	Catalog	Application
TMEM170B	NOVUS Biologicals	NBP2-33739	1:20 for IHC, 1:500 for WB, 1:200 for IF
GSK-3β	Cell Signaling Technology	12456	1:1000 for WB
p-GSK-3β	Cell Signaling Technology	5558	1:1000 for WB
β-catenin	Cell Signaling Technology	8480	1:1000 for WB, 1:100 for IF, 1:100 for IHC, 1:50 for IP
Active β-catenin	Cell Signaling Technology	8814	1:1000 for WB
p-β-catenin(S33/37)	Cell Signaling Technology	2009	1:1000 for WB
IgG Isotype Control	Cell Signaling Technology	3900	1:50 for IP
TCF4	abcam	ab185736	1:1000 for WB
CD44	Cell Signaling Technology	3570	1:1000 for WB
c-myc	Cell Signaling Technology	5605	1:1000 for WB
cyclin D1	Cell Signaling Technology	2922	1:1000 for WB
β-actin	Cell Signaling Technology	4970	1:1000 for WB
Histone H3	Cell Signaling Technology	4499	1:1000 for WB
Sodium Potassium ATPase (Anti-alpha 1)	abcam	Ab7671	1:250 for IF, 1:500 for WB
alpha Tubulin	abcam	ab7291	1:5000 for WB
